# Association of angiotensin converting enzyme gene insertion/deletion polymorphism and familial hypercholesterolemia in the Saudi population

**DOI:** 10.1186/1476-511X-12-177

**Published:** 2013-12-01

**Authors:** Khalid K Alharbi, Tarek S Kashour, Wejdan Al-Hussaini, May Salem Al-Nbaheen, Sarar Mohamed, Rana MW Hasanato, Waleed Tamimi, Mohammed Yahya Al-Naami, Imran Ali Khan

**Affiliations:** 1 Department of Clinical Laboratory Sciences, College of Applied Medical Sciences, King Saud University, P.O. Box 10219, Riyadh 11433, Kingdom of Saudi Arabia; 2 Department of Cardiac Sciences, College of medicine, King Fahad Cardiac Center, King Saud University, P.O. Box 7805 (92), Riyadh 11472, Kingdom of Saudi Arabia; 3 Stem Cell Units, Anatomy Department, College of Medicine, King Khalid University Hospital, Riyadh, Kingdom of Saudi Arabia; 4 Prepratory Year-Saudi Electronic University, Riyadh, Saudi Arabia; 5 Department of Pediatrics, King Khalid University Hospital, College of Medicine, King Saud University, P.O. Box 2925, Riyadh 11461, Kingdom of Saudi Arabia; 6 Department of Pathology, College of Medicine, King Saud University, King Khalid University Hospital, P.O. Box 66533, Riyadh 11586, Kingdom of Saudi Arabia; 7 Department of Pathology & Laboratory Medicine, King Fahad National Guard Hospital, King Khalid University Hospital, College of Medicine King Saud Bin Abdulaziz University for Health Sciences, P.O. Box 22490, Riyadh 11426, Saudi Arabia; 8 Department of Surgery#37, College of Medicine, King Saud University and Affiliated Hospitals, P.O. Box-7805, Riyadh 11472, Kingdom of Saudi Arabia

**Keywords:** Familial hypercholesterolemia, Angiotensin converting enzyme, Polymorphism

## Abstract

**Background:**

The study of the association between genotype and phenotype is of great importance for the prediction of multiple diseases and pathophysiological conditions. The relationship between angiotensin converting enzyme (*ACE*) Insertion/Deletion (I/D) polymorphism and Familial Hypercholesterolemia (FH) has been not fully investigated in all the ethnicities. In this study we sought to determine the frequency of I/D polymorphism genotypes of *ACE* gene in Saudi patients with FH.

**Results:**

This is a case–control study carried out purely in Saudi population. Genomic DNA was isolated from 128 subjects who have participated in this study. *ACE* gene I/D polymorphism was analyzed by polymerase chain reaction in 64 FH cases and 64 healthy controls. There was no statistically significant difference between the groups with respect to genotype distribution. Furthermore, we did not find any significant difference in the frequency of *ACE* I/D polymorphism in FH subjects when stratified by gender (*p* = 0.43).

**Conclusion:**

Our data suggest that *ACE* gene I/D polymorphism examined in this study has no role in predicting the occurrence and diagnosis of FH.

## Introduction

Familial hypercholesterolemia (FH, OMIM 143890) is an inherited disorder of lipoprotein metabolism, transmitted in an autosomal dominant manner and clinically characterized by elevated serum levels of total and low-density lipoprotein (LDL) cholesterol, the presence of tendon xanthomas and premature atherosclerosis [1]. The genetic basis of FH is a large array of mutations in the LDL-receptor (LDLR) gene (OMIM 606945), resulting in a lack of functional receptors for LDL on the liver cell surface, giving rise to increased plasma LDL levels [2]. Population frequency of heterozygous familial hypercholesterolemia (hFH) is approximately 1/500. Although genetically the disease is caused by mutations in the LDLR gene, the clinical phenotype of FH can vary regardless of the mutation and this variability is assumed to be due to both environmental and genetic factors [3]. The clinical phenotype caused by mutations in LDLR, APOB or PCSK9 and characterized by elevated levels of plasma LDL-Cholesterol (LDL-C) is currently referred to as Autosomal Dominant Hypercholesterolemia (ADH, OMIM 603776) [1].

Gene polymorphisms are markers of biologic diversity, and some genotypic variations correlate with specific phenotypes relevant to the human diseases. However, it is not clear whether many of these variants are involved in the pathogenesis of diseases or are merely in proximity to other pathogenic genetic factors-a phenomenon known as linkage disequilibrium. Angiotensin-converting enzyme (ACE) gene insertion/deletion (I/D) polymorphism is responsible for inter individual differences in ACE plasma levels. It is known that the frequency of ACE D alleles varies in different ethnic groups [4]. A previous study by O'Malley et al. [5] have shown an association with FH patients who have myocardial infarction (MI) and coronary heart disease (CHD) [5]. I/D polymorphism have also been associated with risk of MI [6]. ACE gene is involved in the conversion of Angiotensin I to Angiotensin II by its metalloproteinase enzymatic activity. It plays a major role in the rennin–angiotensin (RAS) system. I/D polymorphism (Consisting of a 287-bp fragment) in intron 16 of the gene has been related to the amount of circulating enzyme. Individuals homozygous for the deletion have an approximately two-fold higher level of circulating enzyme in comparison to individuals homozygous for the insertion [7]. Angiotensin II increases vascular smooth muscle cell proliferation, monocyte adhesion, platelet adhesion and aggregation. ACE genotype acting through the tissue or possible plasma ACE concentrations has been implicated in the aetiology of the disease [8].

ACE is a zinc metalloprotease that is widely distributed on the surface of epithelial and endothelial cells. Its gene consists of 26 exons and spans 21 kb on the human chromosome 17. The sequence codes a 1306-amino-acid protein, including a signal peptide. It has a common polymorphism that consists of the presence (I allele) or the absence (D allele) of a 287-bp Alu repeat sequence within intron 16. Although the I/D polymorphism is in the intronic region of the ACE gene, studies have shown that the DD genotype is strongly associated with increased plasma or serum ACE levels [9]. ACE activity in individuals with DD genotype is twice that found in those with II genotypes. Subjects with ID genotype exhibit intermediate levels of ACE [7]. This ACE I/D gene polymorphism has been implicated as a risk factor for a number of pathologies, such as MI, stroke, Type 2 Diabetes Mellitus (T2DM), Diabetic Nephropathy (DN), and hypertension in different ethnic groups, but these findings are far from conclusive [4,10]. The aim of this study was to analyze whether the ACE gene I/D polymorphism is associated with the FH patients in Saudi population.

## Materials and methodology

In this study 64 subjects diagnosed with FH, based on the Deutch working group classification criteria [11]. Patients were recruited from outpatient clinic at King Khalid University Hospital (KKUH), Riyadh. Healthy volunteers (*n* = 64) were recruited from Medical and laboratory staff at KKUH, and individuals attend KKUH for routine checkup and who were not having chronic metabolic/ medical disease. The research protocol was approved by the Institutional Review Board at KKUH (E-12-829). All participants provided written informed consent prior to enrollment into the study.

### Blood

Participants were advised to fast overnight for at least 10 hours prior to sample collection. Five mL of blood sample was collected from every participant; 3 mL of the serum sample was used for biochemical parameters like Total Cholesterol (TC), Triglycerides (TG), HDL-C and LDLC to confirm the FH and 2 mL of EDTA blood sample was used for molecular investigation.

### Biochemical analysis

Serum samples of TC and TG were measured by the standard enzymatic method (Pars Azmon kit, Iran) using an automated RA-1000 (Technicon, USA). Serum LDL-C and HDL-C levels were measured using commercially available enzyme assay kits (Pars Azmon kit, Iran).

### Genomic DNA isolation and genotyping of ACE I/D polymorphism

**Figure 1:**
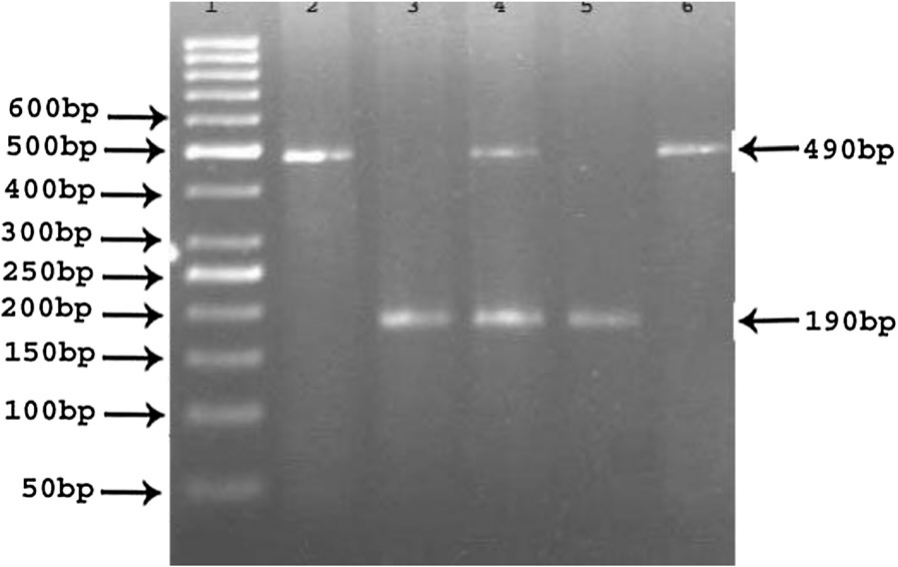
**Identification of ACE I/D polymorphism in 2.5% agarose gel electrophoresis.** Legend: Lane 1: 50bp DNA Marker/Ladder. Lane 2&6: Homozygous II genotype (490 bp). Lane 3&5: Homozygous DD genotype (190 bp). Lane 4: Heterozygous ID genotype (490/190 bp).

The blood samples which were collected in the EDTA tubes were used for extraction of DNA from the peripheral blood leukocytes. Genomic DNA was extracted by standard protocol [12] with Norgen DNA extraction kit (Norgen Biotek corp, Canada). Extracted DNA was quantified by NanoDrop to the DNA concentration. ACE genotypes were determined by Polymerase Chain reaction (PCR) analysis as described in our previous work [12]. Specific oligonucleotide primers: forward primer of 5′CTGGAGACCACTCCCATCCTTTCT-3′ and the reverse primer of 5′- GATGTGGCCATCACATTCGTCACGAT-3′ were applied to acquire a 490 bp DNA fragment in intron 16. The amplification was carried out in a total volume of 20 μL reaction mixture contain 10 μM of each primer, 6 μL of sterile water and 10 μL of 2X master mix which includes MgCl_2_, 10x PCR buffer, dNTPs, 10 units of Taq DNA polymerase (Norgen Biotek corp, Canada) and the 60 ng template DNA. The PCR was performed in a thermal cycler (Applied Biosystems, Hercules, California, USA). The cycling and amplification conditions were as follows; an initial denaturation was set up for 5 minutes at 95°C followed by 35 cycles of denaturation for 30 seconds at 95°C, annealing for 30 seconds at 59°C, extension for 45 seconds at 72°C and the final extension was at 72°C for 5 minutes. The insertion band is considered as 490 bp which represents the I allele and 190 bp is identified as deletion band (without 287-bp Alu insertion) which represents the D allele and the I/D band was 490/190 bp which is considered as the heterozygous (Figure 1). The amplified PCR products were clearly separated on 2.5% agarose gel with ethidium bromide stained (Cambrex, East Rutherford, NJ, USA) and visualized on a transilluminator (Dafco, USA).

### Statistical analysis

All the statistical analyses were carried out using SPSS (Chicago, IL, USA) software version 19.0 for Microsoft Windows®. Clinical characteristics of all subjects were expressed as mean ± SD. Continuous variables were compared between the groups using two-tailed student’s *t* test. Significant cutoff was set at 0.05. Crude ORs with 95% CIs were used to evaluate the strength of the association between ACE I/D polymorphism and FH. Pooled ORs were calculated for allelic contrast (D vs. I), dominant (DD + DI vs. II) and recessive (DD vs. ID + II) genetic model. Z-test was used to determine the significance of the pooled ORs, and *p* value < 0.05 was considered significant. The effect of I/D genotypes of ACE gene was analyzed using general linear model ANOVA for clinical characteristics. A level of *p* <0.05 was considered statistically significant.

## Result

The demographic and the biological data of all the patients participating in this study are shown in Table 1. The lipid profile parameters used in this study are fasting TC, TG, HDL-C and LDL-C. TG, TC and HDL-C levels in the FH group were 1.3 ± 0.50 mmol/L, 6.4 ± 0.68 mmol/L and 1.4 ± 0. 24 mmol/L compared to 1.1 ± 0.36 mmol/L, 4.6 ± 0.39 mmol/L and 0.9 ± 0.21 mmol/L in the control group (*p* < 0.05). The LDL-C level was 4.8 ± 0.55 mmol/L in FH individuals compared to 3.1 ± 0.32 mmol/L in the control group (*p* > 0.05). There was significant difference in the Age, TG, TC and LDL-C levels between the FH subjects and the healthy controls (*p* < 0.05), but there was no difference between FH subjects and healthy controls in HDL-C and gender (*p* > 0.05).

**Table 1:** Demographic characteristics and biochemical profile of FH patients and healthy control

**S. no**		**FH cases**	**Healthy controls**	***p *** **value**
		**(*n*** **= 64)**	**(*n*** **= 64)**	
1	Age (Years)	52.06 ± 10.27	44.8 ± 6.87	*p* = 0.0001
2	Gender: Male/Female	20:44	22:42	*p* = 0.7
3	TG (mmol/L)	1.3 ± 0.50	1.1 ± 0.36	*p* = 0.01
4	TC (mmol/L)	6.4 ± 0.68	4.6 ± 0.39	*p* = 0.04
5	HDL-C (mmol/L)	1.4 ± 0.24	0.9 ± 0.21	*p* = 0.55
6	LDL-C (mmol/L)	4.8 ± 0.55	3.1 ± 0.32	*p* = 0.01

### Screening of I/D polymorphism in the ACE gene

The distribution of ACE I/D polymorphism among FH individuals is shown in Table 2. The frequencies of ACE DD, ID and II genotypes among FH patients were 48.4%, 32.8% and 18.7% respectively. The percentage of D allele was 0.65% and of that of I allele was 0.35%. In the control subjects, the distribution of ACE DD, ID and II genotypes was 39%, 32.9% and 28.1% respectively. The allele frequencies was 0.55% and 0.45% for the D and I alleles, respectively. When we compared the frequency of ACE DD genotypes and D allele in FH cases with healthy controls, the difference was not statistically significant (OR = 1.696, *p* = 0.21 (95% CI = 0.7385-3.893) and OR = 1.481, *p* = 0.12 (95% CI = 0.8953-2.449) (Table 3). Similarly, we did not find any significant difference in the D allele frequencies between patients and controls (Table 3). We also explored the association between ACE I/D polymorphism and FH according to gender and found no significant difference, OR = 0.7373, *p* = 0.43 (95% CI = 0.3403-1.597) (Table 4).

**Table 2:** Genotype and allele frequency of ACE gene

**Genotypes and Alleles**	**FH cases (*n*** **= 64)**	**Healthy controls (*n*** **= 64)**
II	12 (18.7)	18 (28.1)
ID	21 (32.8)	21 (32.9)
DD	31 (48.4)	25 (39)
I	45 (0.35)	57 (0.45)
D	83 (0.65)	71 (0.55)

**Table 3:** Statistical analysis for FH individuals

**S. no**	**Genotypes**	**Odds ratio**
1	DD Vs ID + II	OR-1.465;95% CI = 0.7265–2.956; *p* = 0.28
2	ID + DD Vs II	OR-1.696;95% CI = 0.7385–3.893; *p* = 0.21
3	ID Vs II + DD	OR-1.00;95% CI = 0.4781–2.091; *p* = 0.85
4	D Vs I	OR-1.481;95% CI = 0.8953–2.449; *p* = 0.12

**Table 4:** Genotype and allele frequency of ACE gene between males and females

**Genotypes and alleles**	**Males (*n*** **= 20)**	**Females (*n*** **= 44)**	**OR (95% CI)**	***p *** **value**
II	4 (20%)	8 (18.2%)	0.6087 (0.2083–1.779)	*p* = 0.36
ID	8 (40%)	13 (29.5%)	1.59 (0.5629–4.797)	*p* = 0.40
DD	8 (40%)	23 (52.3%)	0.8889 (0.2335–3.384)	*p* = 0.86
I	16 (0.4)	29 (0.33)	Ref	
D	24 (0.6)	59 (0.67)	0.7373 (0.3403–1.597)	*p* = 0.43

## Discussion

To the best of our knowledge this is the first study to investigate the association of ACE gene I/D polymorphism and FH in Saudi population. In our study FH individuals tended to have higher DD (48.4%) genotypes and more ID (32.9%) genotypes than controls. However, there was no significant difference in the allelic frequency between the patients and controls.

Previous studies addressing the association between FH and ACE gene polymorphism focused on the potential risk of CHD coronary heart disease associated with ACE gene polymorphism in FH patients. For example, O’Malley et al. [5] showed that in heterozygote FH patients with ACE-DD genotype, the incidence of MI is 2.5 times higher and CHD is 2.2 higher than in those with DI or II genotypes. On the other hand, another study [13], did not find any association between ACE gene deletion/insertion polymorphism and increased risk of CHD in patients with FH.

A few studies have been conducted in Saudi Arabia and Middle East looking at the association between ACE gene polymorphism and certain diseases. We have reported previously an association between ACE gene polymorphism and G6PD deficiency [12]. El-Hazmi et al. [14] also showed a significant association between ACE DD genotype and obesity among Saudi patients.

In contrary to that, Dzimiri et al. [15] showed no association between ACE genotype and risk of coronary artery disease in Saudi patients. Another study reported by Chmaisse et al. [16] showed no association between T2DM and ACE gene polymorphism in s mall Lebanese cohort. Our study is limited by small sample size and the fact that our patients have no documented coronary heart disease which may explain our inability to show an association between ACE gene polymorphism and FH.

## Conclusion

In conclusion, our study has not shown an association between I/D polymorphism of ACE gene and FH in the Saudi populations. However, considering the relatively small sample sizes of our study, a firm conclusion cannot be made and larger studies in more well-characterized subjects should be conducted in the future.

## Competing interests

The authors declare that they have no competing interests.

## Authors’ contributions

AKK participated in the design of the study and main investigator of this study. KTS has confirmed the FH patients and drafted the manuscript. AW has carried out the DNA isolation. AMS was the Co-I of this study. SM was an endocrinologist, helped in the FH cases, reviewed and edited the manuscript. HRMW carried out the lipid profile analysis. AMY has collected the control samples. TW has helped in this project with statistics. IAK: Design the study, performed the genotyping, written and edited the manuscript. All authors read and approved the final manuscript.
